# A Way to Improve Adolescents’ Life Satisfaction: School Altruistic Group Games

**DOI:** 10.3389/fpsyg.2021.533603

**Published:** 2021-03-04

**Authors:** Caixia Lu, Lichan Liang, Wenting Chen, Yufang Bian

**Affiliations:** ^1^Collaborative Innovation Center of Assessment Toward Basic Education Quality, Beijing Normal University, Beijing, China; ^2^Faculty of English, Capital Normal University, Beijing, China; ^3^Child and Family Education Research Center, Beijing Normal University, Beijing, China; ^4^Institute of Mental Health and Education, Beijing Normal University, Beijing, China

**Keywords:** altruism, life satisfaction, emotion, school group games, adolescents

## Abstract

Previous studies have shown that adolescents are experiencing growing pains due to their unbalanced physical and mental development. Their life satisfaction showed a steady downward trend with age. Altruism may be an effective way to improve their life satisfaction. Against this background, the current study carried out school altruistic group games (SAGGs) for the first time to explore the role of altruistic group activities in the school context in improving adolescents’ life satisfaction. There were 176 adolescents in the study, including 90 in the experimental group and 86 in the control group, who were enrolled from a junior high school in East China. A 10-week school altruism group game was carried out for the experimental group. The participants in the control group participated in activities that were not related to altruism. Participants in both groups reported their life satisfaction and emotions before and after the games. The findings of this study were as follows: (1) SAGGs can effectively improve adolescents’ life satisfaction, especially school satisfaction; (2) SAGGs can significantly improve adolescents’ emotional state; that is, SAGGs can enhance positive emotions and reduce negative emotions; and (3) SAGGs have different effects on the life satisfaction of adolescents with different initial emotional states. The results of this study not only enrich the existing literature but also provide enlightenment and a reference for schools to improve adolescents’ life satisfaction.

## Introduction

### Life Satisfaction, Altruism, and Emotions

Life satisfaction is an overall cognitive assessment of one’s life conditions most of the time or for a certain period of time according to the criteria of one’s own choice (Diener, 1984). When living conditions meet the living standards set by individuals, they will be satisfied with life. Life satisfaction is an important parameter to measure the quality of life in a society, including domain satisfaction and overall satisfaction. Domain satisfaction refers to the specific evaluation of life domains that have an important influence on individuals, such as family, school, society, etc. Overall satisfaction is an individual’s overall evaluation of their quality of life on the basis of domain evaluation (Zhang et al., 2004).

A previous study found that people’s life satisfaction in adolescence showed a steady downward trend (Wang and Zhang, 2012; Li and Bian, 2016; Lu et al., 2019a). As they grow older, the development and improvement of their cognitive level, the enhancement of self-consciousness, the confusion of role identification, the change of social expectation, and the awakening of sexual consciousness all pose great challenges to individual adaptation. They begin to question and think critically about the people and things around them. Their emotional dependence on their parents and teachers is gradually decreasing and they are seeking independence. They have gradually moved from family to society and have experienced various conflicts and contradictions in the wider world. This imbalance and contradiction of physical and mental development easily leads adolescents to have more emotional and psychological problems and generally shows an increasing trend with age (Zhang and Yu, 2004). Moreover, these negative emotional experiences and psychological problems are negatively correlated with life satisfaction (Panova and Lleras, 2015; Franke et al., 2017). Therefore, the issue of how to improve adolescents’ life satisfaction is an urgent problem to be solved. Previous studies have shown that altruism may be an effective way to improve life satisfaction (Lu et al., 2019b).

Altruism explains why people voluntarily sacrifice their time, money, and even their kidneys to help strangers (Einolf, 2010; Kurleto et al., 2020). Altruism is a long-lasting tendency to help non-relatives without any material return, such as volunteer service and charitable donation (Andreoni, 1990). Altruism is concerned with the well-being of others (Oliner and Oliner, 1988). On the basis of previous studies, Lu et al. (2019b) pointed out that scholars in both the West and the East agreed that altruism includes the following three core ideas: first, altruists hope to increase the well-being of others; second, altruists do not ask for any return, and their altruistic behavior is entirely voluntary; and third, altruism includes four elements: social responsibility, interpersonal trust, loyalty, and sociality, which are consistent in different situations (Rushton et al., 1981; Bartczak, 2015; Lu et al., 2019b; Kurleto et al., 2020).

First, social responsibility is an important factor in altruism. Altruists are more concerned with social relations and ecological issues (Leary et al., 2008). Previous studies have shown that social responsibility is positively correlated with life satisfaction (White, 2009) and can significantly predict life satisfaction and positive emotions (Lu et al., 2019). Chan et al. (2014) found that civic engagement in adolescence is related to higher life satisfaction and lower rates of arrest in emerging adulthood. Citizen participation is one of the forms of social responsibility. Newman et al. (1985) organized 55- to 85-year-old people to carry out social responsibility activities (e.g., volunteer activities in schools). Volunteer activities can effectively improve elderly people’s life satisfaction. In the activities, they find meaning and value in life and can overcome personal trauma in the process of helping others.

The second element of altruism is interpersonal trust (Evans et al., 2013). Previous studies have found that interpersonal trust is a powerful predictor of life satisfaction. The higher the level of interpersonal trust is, the higher the life satisfaction and positive emotions are, and the lower the negative emotions are (Brehm and Rahn, 1997; Jovanoviæ, 2016). A low level of interpersonal trust was associated with a low level of life satisfaction (Yamaoka, 2008). Habibov and Afandi (2015) discussed the changes and interactions of life satisfaction and trust in countries in transition during the global economic and financial crisis in 2007. Their research found that interpersonal trust is a powerful resource that can directly improve life satisfaction in the transitional period. Most importantly, these resources can be used to mitigate the consequences of the crisis. Interpersonal trust can improve life satisfaction. Chinese scholars have obtained similar results (Pei, 2010; Feng and Chi, 2013). However, contemporary adolescents are experiencing interpersonal trust crises, which lead to emotional problems such as hostility, sadness, depression, anxiety, and lack of sense of security (Chen, 2011). These problems are not only not conducive to the formation of young people’s interpersonal communication ability and sound personality but also negatively affect their satisfaction with life. Wang and Chen (2012) investigated the emotional and trust levels of Taiwanese and Hong Kong students in the same course of interactive cooperative learning. The results showed that although they said they trusted each other verbally, when they encountered emotional problems (depression, insecurity, and anger), more students solved them by themselves rather than with “trusted peers.” Therefore, it is urgent to increase interpersonal trust, reduce negative emotions, and improve life satisfaction.

The third element of altruism is empathy (Morelli, 2012; Davis, 2015). Empathy is the ability of individuals to understand and share others’ feelings and respond appropriately to others’ situations (Bloom, 2017). It is found that high-empathy individuals can be more sensitive to the feelings and needs of other individuals, and the other party will also feel that they are understood and accepted, thus generating gratitude. This encourages them to communicate and interact more amicably, to experience happiness and positive emotions for each other, and to enhance life satisfaction. Individuals who often show friendliness to others have more friends (Kardos et al., 2017; Overgaauw et al., 2017), higher life satisfaction, and lower negative emotions (Wei et al., 2011).

Finally, sociality is the fourth element of altruism (Bartczak, 2015; Kurleto et al., 2020). Sociality is the tendency to enjoy getting along with others rather than being alone (Cheek and Buss, 1981). Sociality, which meets the needs of interpersonal communication, is an indispensable part of social life. It has the functions of transmitting information, regulating emotions and behaviors, and providing mental health care (Bu, 2001). As a member of the social activity group, individuals can obtain a sense of identity, belonging, and self-efficacy in the process of communicating with others, which makes it easy for them to better integrate into social groups. Social interaction also helps people’s subjective consciousness change from negative to positive, which makes their spiritual world transition from poverty to richness and causes them to experience more positive emotions and fewer negative emotions (Li and Li, 2016). Sociality promotes higher life satisfaction (Kekkonen et al., 2020). Kafetsios et al. (2017) found that participants in face-to-face social interactions had more positive emotions, fewer negative emotions, and higher life satisfaction than those who interacted on the Internet.

### The Present Study

It is widely known that improving adolescents’ life satisfaction is very important. However, previous studies have mainly focused on people’s life satisfaction, what factors influence their life satisfaction and the internal mechanism with other variables (Heo et al., 2020; Holstein et al., 2020; Lu et al., 2019b). How to improve adolescents’ life satisfaction is an urgent problem to be solved. According to previous studies, altruism may be an effective way to improve adolescents’ life satisfaction. Lu et al. (2019b) found that altruism was a significant predictor of adolescents’ life satisfaction. In addition, with more positive emotions and fewer negative emotions, they will have higher life satisfaction. However, it is not known whether these findings can be used to improve adolescents’ life satisfaction in the real world.

Based on previous theoretical and empirical studies, the present study was the first attempt to carry out school altruistic group games (SAGGs) to explore the role of altruism in improving adolescents’ life satisfaction in the real world. SAGGs are a purposeful, planned, and organized group game based on the class to help participants observe, learn, experience, and practice social responsibility, empathy, sociality, and interpersonal trust, which were factors of altruism, so as to promote their life satisfaction.

We try to answer the following questions:

(1) Can school altruistic games help improve adolescents’ life satisfaction?
(2) Can school altruistic games effectively improve adolescents’ emotional state, that is, enhance positive emotions and reduce negative emotions?
(3) Do school altruistic games have different effects on improving the life satisfaction of adolescents with different emotional states? If so, what are the differences?

Answering these questions cannot only enrich the existing theoretical research but also make a positive contribution to the improvement of adolescents’ life satisfaction. In addition, it is hoped that the findings of this study can provide enlightenment for schools on how to promote better and faster adolescent development.

## Method

### Participants and Procedures

We randomly selected classes from a junior high school in East China and took the classes as a unit to carry on the intervention activities. There were 176 7th-grade participants (89 boys and 87 girls; mean age = 13.41 years). The participants were randomly divided into two groups: 90 adolescents were in the experimental group, and another 86 were in the control group. The ethnicity of the participants in the two groups was Han. Boys and girls accounted for 50%, respectively in the experiment group. In the control group, boys and girls amounted to 51 and 49%, respectively. There was no significant difference in gender between experiment group and control group. Moreover, there was also no significant difference in age between the two groups (*M*_1_ ± *SD*_1_ = 13.34 ± 0.56; *M*_2_ ± *SD*_2_ = 13.49 ± 0.63).

In this study, a double-blind experiment was used. Neither the subject nor the participants knew what the real purpose of the experiment was but were told that these were general group activities. In fact, for participants in the experimental group, the school altruism intervention was carefully designed. The participants in the control group participated in activities that were not related to altruism according to the school schedule, such as “Decoding Your Attention.” The participants did not know whether they were in the experimental group or the control group. The experimenters held masters and doctorate degrees in psychology.

The specific operation procedures of the experiment were as follows. First, before the experiment, the participants in the experimental group and the control group reported life satisfaction and emotional state. Second, the experimental group participated in the intervention experiment for 10 weeks. Third, after the experiment, the two groups reported life satisfaction and emotional state again. Before and after the experiment, the participants completed the questionnaire in the classroom, approximately 15–20 min each time. The participants were willing to participate, and they could choose to withdraw from the study at any time without consequences. SPSS18.0 and Amos17.0 were used for data analysis. Some participants’ data were regarded as invalid data, such as those who did not complete the questionnaire or dropped out of the experiment half-way through. These observations were not included in the final effective data analysis.

### Measures

#### Multidimensional Students’ Life Satisfaction Scale

The level of participants’ life satisfaction was measured using the Multidimensional Students’ Life Satisfaction Scale (Huebner, 1994), which contains five domains of life satisfaction: family, friends, school, living environment, and self. The scale has shown high reliability and validity in Western populations (Greenspoon and Saklofske, 1997; Huebner et al., 1998; Irmak and Kuruüzüm, 2009). The applicability of life satisfaction scale in China may be influenced by culture. Therefore, based on cultural differences between the West and the East, some items of the scale were revised by Chinese scholars (e.g., Zhang et al., 2004). The revised life satisfaction scale includes six subscales: friendship (seven items, e.g., “I have many friends” “My friends will help me when I am in trouble”); family (five items, e.g., “I have a happy family” “My family members get along very well with each other”); academic (five items, e.g., “I am satisfied with my academic achievements”); freedom (four items, e.g., “Basically, I can act freely according to my own thoughts”); school (six items, e.g., “I like to go to school”); and social (six items, e.g., “I live in a place with good social order”). Participants respond to each item on a seven-point Likert scale (1 = strongly disagree to 7 = strongly agree). The total score of general life satisfaction is the sum of friendship, family, academic, freedom, school, and social satisfaction. The score of each dimension such as friendship satisfaction is equal to the total score of each item. The higher the score, the higher the life satisfaction.

Previous studies have shown that the revised life satisfaction scale is suitable for Chinese application and has good reliability and validity (Zhang et al., 2004; Zhang and Zheng, 2005; Lu et al., 2019a). In this study, Cronbach’s *a* was 0.75 for friendship, 0.70 for family, 0.75 for academic, 0.71 for freedom, 0.71 for school, and 0.79 for social. To assess model fit, we used the following indexes: the Chi square goodness of fit test, the Comparative fit index (CFI), the Tucker-Lewis index (TLI), and the Root mean square error of approximation (RMSEA). A non-significant Chi square value means that a model fits the data. Statisticians suggest that when the CFI and TLI are greater than 0.90, the RMSEA is lower than 0.08 and *x^2^/df* is below 3.00 (Hu and Bentler, 1999), the fit of a model is acceptable. The model of life satisfaction used in this study shows an acceptable fit to the data: *x^2^/df* = 1.03, *CFI* = 0.99, *TLI* = 0.99, and *RMSEA* = 0.01.

#### Happiness Scale

The participants’ emotional state was measured using the Happiness Scale (Diener, 1984). The modified Chinese version of the Happiness Scale has been used to assess adolescents’ emotions (Zhang et al., 2004; Liu and Xu, 2015). The revised scale measures two subscales of emotions: positive emotions (six items: e.g., “happy,” “proud,” and “excited”) and negative emotions (eight items: e.g., “sad,” “ashamed,” and “angry”). Adolescents rate the extent to which they experience items related to each emotion on a seven-point scale (1 = “not at all” to 7 = “all the time”). The score of positive/negative emotions was the sum of its six/eight items, respectively. In this study, Cronbach’s *a* was measured as 0.82 for positive emotions and the fitting indexes of structure validity were calculated as follows: *x^2^/df* = 1.77, *CFI* = 0.96, *TLI* = 0.95, and *RMSEA* = 0.06. Cronbach’s *a* was measured as 0.75 for negative emotions and the model shows an acceptable fit to the data: *x^2^/df* = 1.33, *CFI* = 0.98, *TLI* = 0.97, and *RMSEA* = 0.04.

#### Design and Development of the SAGGs

There were five themes in the SAGGs: social responsibility, interpersonal trust, sociality, empathy, and negative emotion regulation. The first four themes are the four elements of altruism (Rushton et al., 1981; Bartczak, 2015; Lu et al., 2019b; Kurleto et al., 2020). Each theme lasted for 2 weeks. There was a different form of group game every week, and each game lasted approximately 90 min. The experimental design was based on the physical and mental development characteristics of junior high school students and typical group activities related to the theme. After the design was completed, front-line teachers, principals, and experts in pedagogy and psychology were invited to review, revise, and improve the design.

As shown in Table 1, the first theme of the SAGGs was social responsibility. In the first week, adolescents learned the story of Yuan Longping, the father of hybrid rice. He is known for his invention of hybrid rice, which solved the problem of food shortages in the early days of the founding of China. Through the study of Yuan Longping’s deeds, we can help students understand that people should not only live for themselves but also strive for others, society, and even the better life of all humankind.

**TABLE 1:** The Intervention program of experimental group.

Themes	Weeks	Contents
Social responsibility	The first week	**Role Model** 1. Profile: Yuan Longping is the first winner of China’s highest science and Technology Award and the father of hybrid rice. 2. Watching: the movie of *Yuan Longping*. 3. Discussion: (1) What made Yuan Longping devote his whole life to rice research and development? (2) What spiritual qualities have you learned from him? (3)What kind of person are you going to be in the future?
	The second week	**Social Practice** Leading students to actively participate in social practice activities: participate in volunteer activities. For example, donate to orphans and the elderly. Go to the nursing home to clean up, perform programs and tell stories for the elderly.
Interpersonal trust	The third week	**Trust Falls** 1. Playing the game When one member of the group fell back straight from the 1.4-meter platform, the others at the bottom of the stage held up a “human bed” with their hands to catch participant who fell off the stage. Everyone in this group should fall off the platform in rotation. 2. Discussion: Ask the participants to talk about their feelings after playing the game of “trust falls.”
	The fourth week	**The Blind and Crutches** 1. Playing the game The game is played in pairs. One plays the blind person and the other the crutch. “Crutches” need to take the blind player over many obstacles to reach the end. 2. Discussion The participants talk about their feelings after playing the game of “blind and crutches.”
Sociality	The fifth week	**Pyramid of name** 1. Playing the game Each participant introduces himself and his personality, hobbies, attitudes and values to open the door for others to understand him. Everyone should try to remember the characteristics of the other members of the group. 2. Discussion (1) How do you feel when someone says your name and characteristics exactly? (2) How do you feel when others can’t name you?
	The sixth week	**Love at the fingertips** 1. Playing the game Each participant expresses whether they are willing to make friends with each other by stretching out one, two, three or four fingers to feel the pleasure of making friends. 2. Discussion (1) How many times did you just hold out your fingers? (2) How many handshakes and hugs you have been completed? (3) Why can you accomplish so much? or why only so little? (4) How do you feel when you see someone holding out more fingers than you? (5) How do you feel when you stretch out more fingers than others?
Empathy	The seventh week	**Approaching left behind children** 1. Show the pictures and videos of the left behind children and tell their stories. 2. Discussion (1) How do you feel after knowing their stories? (2) What do you want to say/do to them? (3) How will you treat your classmates and friends who have difficulties in daily life in the future?
	The eighth week	**Approaching disabled children** 1. Watch the movie *Wonder* 2. Discussion (1) At the beginning, as a person with facial defects, how does Auggie feel? (2) What made him change his attitude toward himself and others? (3) How will you treat your classmates and friends with defects in daily life?
Negative Emotion Regulation	The ninth week	**To be the master of emotions** 1. Help the participants identify negative emotions and their harm. Let them master the ways to regulate negative emotions, such as meditation, talking, exercise, etc. 2. Lead the participants to experience the meditation activity of positive psychological suggestion.
	The tenth week	**Emotional catharsis** The participants were led to the catharsis room to vent their negative emotions.

Young people should contribute to building a better world. The group activities on the theme of social responsibility took a three-step approach. The first step was to ask the participants to collect information about Yuan Longping, the more comprehensive the better, and then introduce it to others. For example, regarding the course of founding and building the new China, the Chinese people wrote the most magnificent chapter on the history of the Chinese nation, and many heroic models emerged. Therefore, the selection of “100 people who have moved China since the founding of new China” was launched. Yuan Longping was selected as one of the 100 people who moved China. He had two dreams: one was “the dream of enjoying the cool under the ears of rice,” which means that the super rice in his experimental field was as high as sorghum. He and his assistant could sit under the ear of rice to enjoy the coolness. His second dream was “the global dream of hybrid rice coverage.” If half of the world’s paddy fields were planted with hybrid rice, grain yield could be increased by 150 million tons. Then, people all over the world would have food to eat, and there would be no hunger. The second step was to watch a movie called Yuan Longping. Through watching movies, adolescents can learn more about Yuan Longping and be infected by his spirit of taking social responsibility. In the second week, we carried out social practice. We organized students to participate in social practice activities, such as volunteer activities of donating clothes and books for orphans and the elderly, cleaning for elderly individuals in nursing homes, performing programs, and bringing warmth and blessing to them.

The second theme was interpersonal trust. The group games of “Trust Falls” and “Blind People and Crutches” were performed to let the adolescents establish mutual trust in the activities of helping each other complete the task together. The first game was “Trust Falls,” which was conducted in the third week. When one member of the group fell back straight from the 1.4-meter platform, the others at the bottom of the stage held up a “human bed” with their hands to catch people who fell off the stage. Everyone in this group should fall off the platform in rotation. After the game, the participants were asked to talk about their feelings, such as “How do you feel when you are about to fall off the platform?” The second game was “Blind People and Crutches,” which was held in the fourth week. The process of the game was as follows: the participants in the experimental group were randomly divided into several groups. Each group was composed of two participants: one was a “blind person,” and the other was a “crutch.” The blind man was blindfolded and turned around three times to temporarily lose his sense of direction. With the help of the “crutches,” the “blind men” cross the obstacles and reach the destination along the route chosen by the experimenters. In this process, “crutches” can only use gestures, movements and other body language to help the “blind” experience various feelings but cannot talk with each other. After arriving at the destination, roles are changed and repeated so that everyone can experience the feeling of being “blind” and “crutches,” and the route changes. Then, the participants talked about their feelings after playing the game.

The third topic was sociality, which aims to increase adolescents’ interest in interpersonal communication. The theme was still made up of two games: one is “Pyramid of Name,” and the other is “Love at the Fingertips.” The process of the “Pyramid of Name” game is as follows: each student introduces himself with a sentence, which must include two pieces of information: name and unique characteristics. For example, “My name is Liu Ming. I am a lively and active boy.” When the second student introduces himself, he must start from the first one, for example, “I’m Zhang San sitting beside lively and active boy Liu Ming. I am an introverted and shy boy,” and the third student says, “I am Lisi sitting next to Zhang San, who is introverted and shy. He is sitting next to Liming, who is lively and active. I like playing basketball.” The last person must add the self-introduction of all the students in front of him. At the end of the game, the experimenter will organize them to have a discussion, such as “How do you feel when the others remember your hobbies.”

The process of the “Love at the Fingertips” game is as follows: students are divided into two equal groups. One group of members forms an inner ring, the others form an outer ring, and members of the inner and outer circles stand face to face. When the master gives the command of “gesture,” each member reaches out 1–4 fingers to each other: (1) stretch out one finger to indicate “I do not want to know you yet”; (2) stretch out two fingers to indicate “I am willing to know you and make a nodding friend with you”; (3) stretch out three fingers to indicate “I’m very happy to meet you and want to know more about you and to be a common friend with you”; (4) hold out four fingers to indicate “I like you very much and want to be good friends with you. I will share happiness and pain with you.” Rules of the game: (1) if the number of fingers extended by two people is not the same, then stand still and do nothing; (2) if both of them hold out one finger, they then turn their faces to their right and stamp their feet heavily; (3) if both of them hold out two fingers, they then smile and nod to each other; (4) if both of them stretch out three fingers, they then smile and shake hands with each other; (5) if two people hold out four fingers, they then embrace each other warmly. Each time a group of “gestures” is completed, the members of the outer circle take a step to the right and stand face to face with the next member, and the follower makes corresponding gestures and actions according to the leader’s command until all the outer and inner circle students have completed a set of “gestures.” Finally, some questions are discussed, such as how many handshakes and hugs you completed.

The fourth theme was empathy. Empathy is the ability to put oneself in others’ shoes to understand their thoughts and feelings (Eisenberg, 2000). In this module, we focused on two types of people. One was left behind children, which refers to children and adolescents under 18 years old who are left behind in their registered residence and cannot live with their parents (Luo et al., 2009; Wei, 2015). The family life of these children is generally difficult, and their parents have to work in large cities far away from home. They can only live with their grandparents or other relatives. Some live in schools. The other was children with physical defects, such as Auggie, the hero in the film “Wonder.” Through the understanding of these two vulnerable groups of children, the participants felt that their lives are not easy and were then willing to help them.

Finally, negative emotion regulation and control training was carried out. First, this approach helped participants learn to identify negative emotions and recognize the harm of negative emotions to their growth. Thus, students mastered the methods of regulating and controlling negative emotions, such as emotional catharsis. The students were led to the catharsis room, where there was a silicone simulation catharsis person present, whom they could beat at will. There are also many types of funny mirrors in the room that showed the strange shape of the students, which made them laugh to dispel the bad mood.

## Results

### Effectiveness Analysis of School Altruistic Group Games on Improving Adolescents’ Life Satisfaction

The life satisfaction scores of the control and experimental groups are shown in Table 2. To explore the effectiveness of altruistic intervention on improving adolescents’ life satisfaction, a repeated measures ANOVA was used to compare the levels of their general life satisfaction, friendship, family, academic, freedom, school, and social satisfaction.

**TABLE 2:** The influence of intervention experiment on life satisfaction.

		Control group (*M* ± *SD*)	Experimental group (*M* ± *SD*)	*MD*
General life satisfaction	1	158.10 ± 9.14	142.2 ± 17.84	15.90 ± 2.12**
	2	153.07 ± 20.05	166.90 ± 25.97	−13.83 ± 3.49**
Friendship satisfaction	1	36.38 ± 3.48	31.87 ± 6.09	4.51 ± 0.74**
	2	34.79 ± 5.99	35.20 ± 7.68	−0.41 ± 1.03
Family satisfaction	1	37.81 ± 4.54	35.06 ± 4.56	2.75 ± 0.68**
	2	36.84 ± 6.82	37.38 ± 6.86	−0.54 ± 1.03
Academic satisfaction	1	26.34 ± 3.46	23.28 ± 5.57	3.06 ± 0.69**
	2	24.72 ± 5.67	28.21 ± 6.86	−3.48 ± 0.95**
Freedom satisfaction	1	24.14 ± 3.13	20.09 ± 5.89	4.05 ± 0.71**
	2	21.69 ± 5.65	23.95 ± 5.95	−2.27 ± 0.87^∗^
School satisfaction	1	25.46 ± 2.93	24.52 ± 4.15	0.93 ± 0.54
	2	27.01 ± 4.23	32.60 ± 5.90	−5.59 ± 0.77**
Social satisfaction	1	7.97 ± 1.83	7.37 ± 2.55	0.59 ± 0.33
	2	8.01 ± 2.35	9.55 ± 2.65	−1.53 ± 0.38**

1 = pre-test; 2 = post-test.

*p < 0.05, **p < 0.01.

For general life satisfaction, the results showed that the main effect of time was significant (*F*_(1,174)_ = 26.03, *p* = 0.001, $\eta_{p}^{2}$ = 0.13). The interaction effect between time and group was significant (*F*_(1,174)_ = 59.49, *p* = 0.001, $\eta_{p}^{2}$ = 0.26). A further simple effect test showed that in the pre-test, the general life satisfaction of the experimental group was significantly lower than that of the control group (*MD* = −15.90, *p* = 0.001). However, in the post-test, the general life satisfaction of the experimental group was significantly higher than that of the control group (*MD* = 13.82, *p* = 0.001). These results showed that the SAGGs effectively improved adolescents’ general life satisfaction in the experimental group.

For friendship, family, academic, freedom, school, and social satisfaction, the results showed that while the main effect of time was significant for academic, school, and social satisfaction (*F*_1(1,174)_ = 9.07, *p*_1_ = 0.003, $\eta_{p}^{2}$ = 0.05; *F*_2(1,174)_ = 107.63, *p*_2_ = 0.001, $\eta_{p}^{2}$ = 0.38; *F*_3(1,174)_ = 21.57, *p*_3_ = 0.001, $\eta_{p}^{2}$ = 0.11), the main effect of time was not significant for friendship, family, and freedom satisfaction (*F*_1(1,174)_ = 2.36, *p*_1_ = 0.126; *F*_2(1,174)_ = 1.25, *p*_2_ = 0.266; *F*_3(1,174)_ = 1.40, *p*_3_ = 0.238). For friendship, family, academic, freedom, school, and social satisfaction, the interaction effects between time and group were significant (*F*_1(1,174)_ = 18.89, *p*_1_ = 0.001, $\eta_{p}^{2}$ = 0.10; *F*_2(1,174_) = 7.32, *p*_2_ = 0.008, $\eta_{p}^{2}$ = 0.04; *F*_3(1,174)_ = 35.58, *p*_3_ = 0.001, $\eta_{p}^{2}$ = 0.17; *F*_4(1,174)_ = 28.37, *p*_4_ = 0.001, $\eta_{p}^{2}$ = 0.14; *F*_5(1,174)_ = 49.36, *p*_5_ = 0.001, $\eta_{p}^{2}$ = 0.22; *F*_6(1,174)_ = 19.88, *p*_6_ = 0.001, $\eta_{p}^{2}$ = 0.10).

The further simple effect test showed that in the pre-test, friendship and family satisfaction levels of the experimental group were significantly lower than those of the control group (*MD*_1_ = −4.50, *p*_1_ = 0.001; *MD*_2_ = −2.75, *p*_2_ = 0.001). In the post-test, friendship and family satisfaction levels of the experimental group were slightly higher than those of the control group, although they were not significant (*MD*_1_ = 0.40, *p*_1_ = 0.694; *MD*_2_ = 0.53, *p*_2_ = 0.602). However, the results also showed that the SAGGs improved friendship and family satisfaction of adolescents to a certain extent. The academic and freedom satisfactions of the experimental group were significantly lower than that of the control group in the pre-test (*MD*_1_ = −3.06, *p*_1_ = 0.001; *MD*_2_ = −4.05, *p*_2_ = 0.001). In the post-test, the academic and freedom satisfactions of the experimental group were significantly higher than those of the control group (*MD*_1_ = 3.48, *p*_1_ = 0.001; *MD*_2_ = 2.26, *p*_2_ = 0.010). There was no significant difference in school and social satisfaction between the control and experimental groups in the pre-test (*MD*_1_ = −0.93, *p*_1_ = 0.086; *MD*_2_ = −0.59, *p*_2_ = 0.076). Alternatively, in the post-test, the school and social satisfaction levels of the experimental group were significantly higher than those of the control group (*MD*_1_ = 5.59, *p*_1_ = 0.001; *MD*_2_ = 1.53, *p*_2_ = 0.001).

### Effectiveness Analysis of School Altruistic Group Games on Improving Adolescents’ Emotions

The positive and negative emotion scores of the control group and experimental group are shown in Table 3. This study compared differences in the emotions of the two groups in the pre-test and post-test using repeated measures ANOVA. For positive and negative emotions, the main effect of time was significant (*F*_1(1,174)_ = 65.41, *p*_1_ = 0.001, $\eta_{p}^{2}$ = 0.27; *F*_2(1,174)_ = 9.07, *p*_2_ = 0.003, $\eta_{p}^{2}$ = 0.05). The interaction effect between time and group was significant (*F*_1(1,174)_ = 17.17, *p*_1_ = 0.001, $\eta_{p}^{2}$ = 0.09; *F*_2(1,174)_ = 4.02, *p*_2_ = 0.046, $\eta_{p}^{2}$ = 0.02). The further simple effect test showed that in the pre-test, positive emotions/negative emotions of the experimental group were significantly weaker/stronger than those of the control group (*MD*_1_ = −2.92, *p*_1_ = 0.001; *MD*_2_ = 3.02, *p*_2_ = 0.003). Alternatively, in the post-test, positive emotions/negative of the experimental group were significantly stronger/weaker than those of the control group (*MD*_1_ = 2.23, *p*_1_ = 0.031; *MD*_2_ = −3.62, *p*_2_ = 0.001).

**TABLE 3:** The influence of intervention experiment on emotions.

		Control group (*M* ± *SD*)	Experimental group (*M* ± *SD*)	*MD*
Positive emotion	1	21.60 ± 4.36	18.67 ± 5.79	2.92 ± 0.77**
	2	24.05 ± 6.17	26.29 ± 7.40	−2.24 ± 1.02^∗^
Negative emotion	1	18.58 ± 6.87	21.60 ± 6.37	−3.02 ± 1.00**
	2	17.87 ± 4.40	14.97 ± 4.94	2.90 ± 0.70**

1 = pre-test; 2 = post-test.

*p < 0.05, **p < 0.01.

### Comparison Among Adolescents in the Experimental Group With Different Initial Levels of Emotion

To further examine the effectiveness and applicability of the SAGGs, a repeated measures ANOVA test was conducted among three groups: adolescents in the experimental group with high, middle, and low initial levels of overall emotional state. The three groups were divided according to the participants’ overall emotional state prior to the experiment. The score of the overall emotional state is equal to the score of positive emotion minus the score of negative emotion. The higher the score, the more positive the participants were. As shown in Table 4, the SAGGs promoted life satisfaction in the three groups to varying degrees.

**TABLE 4:** Comparison among students with different initial levels of emotion in the experimental group.

variable	Source of variation	*SS*	*df*	*MS*	*F*	*p*	$\eta_{p}^{2}$	*Post-hoc*
1	Time	26158.56	1	26158.56	60.22	0.001	0.42	C > A**, C > B**
	Group	5677.15	2	2838.57	5.64	0.005	0.12	
	Time × group	879.38	2	439.69	1.01	0.368		
2	Time	474.59	1	474.59	14.58	0.001	0.15	C > A**, C > B**
	Group	646.95	2	323.47	5.66	0.005	0.12	
	Time × group	77.51	2	38.75	1.19	0.309		
3	Time	233.22	1	233.22	6.23	0.015	0.07	
	Group	110.45	2	55.22	1.79	0.172		
	Time × group	3.77	2	1.89	0.05	0.951		
4	Time	1046.13	1	1046.13	28.86	0.001	0.25	C > A**
	Group	291.46	2	145.73	3.62	0.031	0.08	
	Time × group	1.70	2	0.85	0.02	0.977		
5	Time	636.43	1	636.43	16.72	0.001	0.16	
	Group	145.56	2	72.78	2.39	0.098		
	Time × group	131.81	2	65.90	1.73	0.183		
6	Time	2801.45	1	2801.45	109.86	0.001	0.57	C > A**, C > B**
	Group	181.34	2	90.67	3.65	0.030	0.08	
	Time × group	72.81	2	36.40	1.42	0.246		
7	Time	201.04	1	201.04	34.36	0.001	0.29	
	Group	33.05	2	16.52	2.26	0.110		
	Time × group	24.58	2	12.29	2.10	0.129		

*1 = general life satisfaction; 2 = friendship satisfaction; 3 = family satisfaction; 4 = academic satisfaction; 5 = freedom satisfaction; 6 = school satisfaction; 7 = social satisfaction.*

A = low level of positive emotion group; B = middle level of positive emotion group; C = high level of positive emotion group.

*p < 0.05, **p < 0.01.

As shown in Table 4, the main effect of time on adolescents’ life satisfaction is significant. The post-test scores of general life satisfaction, friendship, family, academic, freedom, school, and social satisfaction of adolescents in the high, medium, and low emotional state groups were significantly higher than those in the pre-test (*MD*_1_ = 24.67, *p*_1_ = 0.001; *MD*_2_ = 3.32, *p*_2_ = 0.001; *MD*_3_ = 2.32, *p*_3_ = 0.015; *MD*_4_ = 4.93, *p*_4_ = 0.001; *MD*_5_ = 3.84, *p*_5_ = 0.001; *MD*_6_ = 8.07, *p*_6_ = 0.001; *MD*_7_ = 2.16, *p*_7_ = 0.001). The results showed that the SAGGs had a significant effect on the improvement of life satisfaction of the three groups of adolescents. In addition, there were significant differences in general life satisfaction, friendship, and academic and school satisfaction among the high, middle, and low emotional state groups. The general life satisfaction of the high-level group was significantly higher than that of the low- and medium-level emotional groups using a *post hoc* test (*MD*_1_ = 12.61, *p*_1_ = 0.004; *MD*_2_ = 11.87, *p*_2_ = 0.006). The friendship and school satisfactions of the high-level group were significantly higher than those of the low- and medium-level groups (*MD*_1_ = 4.27, *p*_1_ = 0.003; *MD*_2_ = 3.99, *p*_2_ = 0.006; *MD*_3_ = 2.38, *p*_3_ = 0.013; *MD*_4_ = 1.93, *p*_4_ = 0.041). For academic satisfaction, the scores of the high-level group were significantly higher than those of the low-level group (*MD* = 3.18, *p* = 0.009). These findings showed that the SAGGs have the strongest effect on adolescents with a high emotional state.

## Discussion

The study is the first attempt to construct altruistic school group games for adolescents to improve their life satisfaction. The results reveal several important insights.

### The Effectiveness of a SAGGs Program in Improving Adolescents’ Life Satisfaction

The study found that SAGGs can significantly improve adolescents’ life satisfaction. This finding not only confirmed the previous theoretical and empirical studies on the relationship between altruism and life satisfaction but also showed the effectiveness of altruistic intervention games. Altruistic group games significantly increased the life satisfaction of adolescents. This may benefit from the following three aspects. First, the design of the experiment was based on the internal mechanism model of the relationship between altruism and life satisfaction, which has a solid theoretical and empirical basis. Based on the four elements of altruism–social responsibility, interpersonal trust, empathy, and sociality–this study aimed to help students feel social responsibility in model learning and volunteer practice, enhance interpersonal trust in group games, train empathy, and increase the fun of interpersonal communication. For example, during the discussion after watching the film Yuan Longping, some adolescents said, “Yuan Longping began to study hybrid rice in the 1960s, which successfully solved China’s food problem and made outstanding contributions to world food security. I want to learn from Yuan Longping, master advanced science and technology, and make contributions to the development of the motherland and all humanity.” Another example is that in the game of the “Blind People and Crutches,” the adolescents had many feelings. A “blind man” said “Although I can’t see anything, I believe my partner very much. I didn’t bump into anything from the beginning to the end of the activity. She led the way very well and was very careful.” One “crutch” said “I was worried that my companion would get hurt, so I always helped her walk very carefully and slowly. I think she believes me, which makes me feel responsible.” In the activity of “Love at the Fingertips,” adolescents felt the pleasure of making friends. They also mastered some skills of interpersonal communication. When adolescents saw the pictures of left-behind children and heard the story of their hard lives, many of them silently shed tears.

Second, the design of the experimental scheme was based on the age characteristics of adolescents’ physical and mental development and group games were used because they are more vivid and interesting. Compared with rigid classroom teaching, such games can better stimulate the curiosity and interest of adolescents. Therefore, young people take an active part in group activities every time. Finally, before the implementation of the experimental program, we widely consulted experts in pedagogy and psychology, teaching researchers, principals, and teachers. They provided valuable comments and constructive suggestions. We studied their comments carefully and improved the program. In addition, in the process of the experiment, we strictly monitored the operation process and further adjusted and improved the plan according to the actual situation. The results showed that altruistic group activities in school may contribute to the improvement of adolescents’ life satisfaction.

### The Effectiveness of a SAGGs Program in Improving Adolescents’ Emotions

Altruists help others with no return, even strangers. Even so, altruists gain an emotional “warm glow” (Andreoni, 1990, p.464) or a “helper high” (Dossey, 2018, p.393) from helping others. This proves an old Chinese proverb: “help others and be happy with yourself.” Yang and Chen (2011) asked if participants would like to donate some money or to work as a volunteer to help earthquake survivors. Participants who wanted to help these survivors reported fewer negative emotions than those who read the same article. Lu et al. (2019b) found that altruism was a significant positive/negative predictor of positive/negative emotions. Previous studies have shown that emotion is an important factor affecting adolescents’ life satisfaction, and positive/negative emotions significantly positively/negatively predict life satisfaction. Data from 23 European countries draw the same conclusion (Mutz and Kämpfer, 2013). Bastian et al. (2014) used the data of more than 9000 students in 47 countries to study the life satisfaction of middle school students in countries that attach great importance to the cultivation of positive emotions. The results show that these countries increase the frequency of students’ positive emotional experiences and then improve their level of life satisfaction. In other words, the more positive emotions middle school students experience, the higher their life satisfaction. In contrast, the fewer positive emotions they experience, the lower their life satisfaction. Therefore, in real life, as the main educational institution for adolescents, can schools improve their emotions and life satisfaction through lively and vivid group altruistic games? The results of this study confirm that altruistic group games in a school can effectively improve their positive emotions and reduce their negative emotions.

Adolescence is a special period in the process of human growth. It is a stage that is full of contradictions, conflicts, and confusion. Their physical growth is rapid, but their mental development is slow. They are eager to mature, but they are incompetent. They are faced with the challenge brought by the imbalance of physical and mental development. The immature development of the prefrontal cortex of the executive control function and imperfect connections to related brain areas easily lead to adolescents experiencing impulsive, irritable, and other negative emotions. In addition, the blind pursuit of scores by parents and the fiercely competitive environment also bring all kinds of pressure to them. These “growing pains” negatively affect life satisfaction (Lu et al., 2019b). Moreover, through the interviews, it was found that compared with the control group, the adolescents in the experimental group had more homework. Their teachers and parents were stricter with their study, and they had little free time to play. They may have experienced more “growing pains.” These factors may be the reasons that the students in the experimental group had more negative baseline emotions than those in the control condition. Therefore, this study carried out group activities of negative emotion regulation to help them correctly understand and identify negative emotions and master the methods and skills of negative emotion regulation and control strategies. Group activities can help to reduce adolescents’ negative emotions. When they came out of the vent room, one student said, “It’s a great feeling. I’ve never been so happy.” The other said, “I feel comfortable.”

### Different Effectiveness of SAGGs on Life Satisfaction of Adolescents With Different Emotional States

To further explore the indirect effect of altruistic group games and to provide more insight into ways of promoting adolescents’ life satisfaction, this study examined the different effects of the intervention on adolescents with different emotional state levels in the experimental group. The participants were divided into three groups: low, middle, and high levels according to their initial emotional state before games. It was found that the SAGGs experiment promoted the life satisfaction levels of the three groups to varying degrees. Moreover, the SAGGs produced the strongest effect on the life satisfaction of adolescents in high-level emotional states. For adolescents with a high level of emotional state, the results of the study indicated the maxim that “a happy person is happier.” People who feel more love and are happier than others are more likely to be kind and generous to others (Emanuel, 2015). The study of donation behavior showed that happier people have a greater tendency to donate (Shankland, 2012). Altruistic individuals receive more rewards than recipients during their altruistic behavior, which can significantly enhance their positive emotion (Schwartz et al., 2013). Therefore, adolescents with a high level of emotional state are more likely to exhibit altruistic behaviors such as donating a kidney (Brethel-Haurwitz and Marsh, 2014), which can in turn contribute to their life satisfaction (Xi et al., 2017).

The implication of this finding is that different interventions should be designed according to the different baseline levels of adolescents’ emotional states. We should treat different groups in different ways and strengthen the interaction between peers. The breadth, depth, content and form of the intervention should therefore be different for groups possessing different emotional state levels. Adolescents with a high level of SWB not only participated in the intervention but also helped to organize and implement the SAGGs program. This mutual education and influence of the participants may yield unexpected results. Furthermore, the depth and breadth of the SAGGs program need to be expanded for adolescents with lower levels of SWB.

### Limitations and Future Directions

Although this study offers several important insights, its limitations should be considered and may provide directions for future research. First, there is a representative problem of sample selection in this study, as the participants in this study were randomly selected from East China with an average age of approximately 13 years old. However, the effect of SAGGs on other areas or other age groups is still unknown. Therefore, we can explore the effect of SAGGs on people in different regions to increase the popularization of SAGGs. Moreover, we should carry out altruistic interventions with senior high school students, college students, and even adults in the future, which may contribute to the construction of a harmonious and symbiotic society. Second, this study is an altruistic game activity carried out in the school environment. The results of this study may be applicable to school situations, but whether these findings can be applied to other situations (such as family or community) is still unknown. In addition, how can families and communities contribute to the improvement of adolescents’ life satisfaction? This is also a problem to be solved in future research. Finally, this study conducted the same altruistic intervention for all participants in the experimental group, regardless of whether their original emotional state was high, medium, or low. The results showed that the SAGGs had different effects on adolescents with different emotional states. However, this study did not explore how to carry out altruistic activities for adolescents with different emotional states to improve their life satisfaction. Therefore, this issue is also a direction of future research.

## Conclusion

This study is the first attempt to explore the role of SAGGs in improving adolescents’ life satisfaction and emotions in the school environment. SAGGs can not only significantly improve adolescents’ life satisfaction but also significantly increase their positive emotions and reduce their negative emotions. These findings not only enrich the existing research system of altruism, life satisfaction, and emotion but also provide an effective way to improve adolescents’ life satisfaction and emotions. In addition, the study confirmed that the SAGGs herein produced different effects on adolescents’ life satisfaction with different emotional states. The SAGGs had the strongest effect on the life satisfaction of adolescents in the high-level group. These findings provide us with enlightenment and a scientific basis for more targeted intervention activities in the future.

## Data Availability Statement

All datasets generated for this study are included in the article/Supplementary Material.

## Ethics Statement

This study was carried out in accordance with the recommendations of the Research Ethics Committee of Beijing Normal University with written informed consent from all participants’ parents/legal guardians, in accordance with the Declaration of Helsinki. The protocol was ethically approved by the Research Ethics Committee of Beijing Normal University. Written informed consent to participate in this study was provided by the participants’ legal guardian/next of kin. Written informed consent was obtained from the individual(s), and minor(s)’ legal guardian/next of kin, for the publication of any potentially identifiable images or data included in this article.

## Author Contributions

YB and LL planned the study. CL and WC collected and analyzed the data, and drafted the manuscript, with critical input from the other authors. All authors approved the final version of the manuscript for submission.

## Conflict of Interest

The authors declare that the research was conducted in the absence of any commercial or financial relationships that could be construed as a potential conflict of interest.
